# Comparing Two Independent Satellite-Based Algorithms for Detecting and Tracking Ash Clouds by Using SEVIRI Sensor

**DOI:** 10.3390/s18020369

**Published:** 2018-01-27

**Authors:** Alfredo Falconieri, Michael C. Cooke, Carolina Filizzola, Francesco Marchese, Nicola Pergola, Valerio Tramutoli

**Affiliations:** 1 Institute of Methodologies for Environmental Analysis (IMAA), Italian Research Council (CNR), 85050 Tito Scalo, Potenza, Italy; carolina.filizzola@imaa.cnr.it (C.F.); francesco.marchese@imaa.cnr.it (F.M.); nicola.pergola@imaa.cnr.it (N.P.); 2 Met Office, FitzRoy Road, Exeter, Devon EX1-3PB, UK; michael.c.cooke@metoffice.gov.uk; 3 School of Engineering, University of Basilicata, Via dell’Ateneo Lucano 10, 85100 Potenza, Italy; valerio.tramutoli@unibas.it

**Keywords:** Eyjafjallajökull, ash clouds, SEVIRI, AIRS, RST_ASH_, London VAAC method

## Abstract

The Eyjafjallajökull (Iceland) volcanic eruption of April–May 2010 caused unprecedented air-traffic disruption in Northern Europe, revealing some important weaknesses of current operational ash-monitoring and forecasting systems and encouraging the improvement of methods and procedures for supporting the activities of Volcanic Ash Advisory Centers (VAACs) better. In this work, we compare two established satellite-based algorithms for ash detection, namely RST_ASH_ and the operational London VAAC method, both exploiting sensor data of the spinning enhanced visible and infrared imager (SEVIRI). We analyze similarities and differences in the identification of ash clouds during the different phases of the Eyjafjallajökull eruption. The work reveals, in some cases, a certain complementary behavior of the two techniques, whose combination might improve the identification of ash-affected areas in specific conditions. This is indicated by the quantitative comparison of the merged SEVIRI ash product, achieved integrating outputs of the RST_ASH_ and London VAAC methods, with independent atmospheric infrared sounder (AIRS) DDA (dust-detection algorithm) observations.

## 1. Introduction

On 14 April 2010, an intense phreatomagmatic eruption took place beneath the ice cap near the summit of the Eyjafjallajökull volcano (63°38′ N, 19°36′ W) in Iceland, emitting large quantities of fine ash of trachyandesite composition [1]. Because of the wind direction, the ash plume reaching an altitude of about 5–9 km above sea level moved towards the south-east and, thereafter, over Europe, causing unprecedented air-traffic disruption [1,2]. Several flights were cancelled for safety reasons and millions of passengers were stranded across the world [3]. The economic losses to airlines were enormous, and estimated to be $250 million per day during 15–20 April [4]. On-site observations showed that more than 50% of the emitted solid material had a radius less than 50 μm in diameter, and ≤20% was smaller than 10 μm. The interaction between the magma and water arising from melting glaciers favored the magma fragmentation [2]. In the evening of 18 April, the eruption style changed from phreatomagmatic to magmatic, releasing a lower amount of volcanic ash in the atmosphere [4]. At the beginning of May, the explosive activity renewed and the airborne ash affected Western Europe once again, causing additional airspace closures [5]. On 18 May, the ash plume reached an altitude of 7 km, while in the following days it decreased in height, suggesting a significant reduction of the magma emission rates [1]. 

The Eyjafjallajökull eruption, which ended on 22 May, revealed some important limitations in the operational monitoring and forecasting of ash plumes in Europe [5] and encouraged the development of methods and procedures better able to support the activities of the Volcanic Ash Advisory Centers (VAACs), especially during a strong eruptive crisis. 

Among recent work performed using satellite data, new algorithms of ash detection have been proposed and tested to map and track the presence of volcanic ash in the atmosphere (see Section 2). One of these methods, exploiting the spectral and temporal features of the spinning enhanced visible and infrared imager (SEVIRI) sensor (flying aboard the Meteosat Second Generation (MSG) geostationary satellite) was developed at the London VAAC hosted by the Met Office (UK). The algorithm was first tested studying the Eyjafjallajökull 2010 eruption [6]. Afterwards, it was used to identify and track the ash cloud emitted by the Grímsvötn volcano (Iceland) in May 2011 [7].

In this work, we compare this method to the RST_ASH_ algorithm [8], investigating differences/similarities in the identification of ash clouds emitted by the Eyjafjallajökull volcano. RST_ASH_ is a state-of-the-art technique whose performance was assessed in different geographic areas using both advanced very-high resolution radiometer (AVHRR) and moderate-resolution imaging spectrometer (MODIS) instruments [8,9,10,11]. Recently, RST_ASH_ has been optimized for better discriminating ash from meteorological clouds using SEVIRI data [12].

This study investigates possible advantages arising from the combination of the aforementioned ash-detection methods in an operational context, performing a quantitative comparison of SEVIRI ash maps with independent atmospheric infrared sounder (AIRS) data products. 

The paper is organized as follows: Section 2 reports the scientific background and the state of art of ash detection from space; Section 3 describes the RST_ASH_ and the London VAAC methods; Section 4 details the used data; Section 5 reports the achieved results; and finally, in Section 6 the results and future perspectives of this work are discussed.

## 2. Background

The BTD (brightness temperature difference) method [13] was one of the first and most widely applied satellite-based techniques for ash-cloud detection purposes [14,15,16,17]. The algorithm exploits the reverse absorption behavior shown by silicate particles in the TIR (thermal infrared) region (at 11 µm and 12 µm wavelengths) in comparison with water droplets and ice [18] in order to detect volcanic ash using a fixed-detection threshold on single BTD images (e.g., *BT11 − BT12* < 0). Thanks to easy implementation and a good capacity for detecting airborne ash under specific observational conditions (e.g., semi-transparent clouds, low atmospheric water vapor content, high thermal contrast between ground and top of the plume), the BTD technique has been a useful tool for VAACs [19]. However, in spite of the aforementioned advantages, many factors (e.g., moist atmosphere, thermal-relaxation phenomena, convective clouds overshooting the tropopause, presence of ash over a cold background) widely discussed and debated in scientific literature [20,21,22] may affect its performance. 

In order to improve the identification of volcanic ash by satellites, more sophisticated detection schemes have been proposed. Some authors have developed specific procedures to minimize the impact of atmospheric water vapor on the BTD signal, which may be strongly attenuated or completely masked in moisture-rich environments [23,24]. Other authors have used such additional spectral bands as the visible (VIS) and/or medium infrared (MIR) of sensors like AVHRR and MODIS [8,25,26] to increase the accuracy of ash detection, reducing false positives compared to the traditional BTD method. Among the recent ash-detection techniques, some authors have used absorption optical depth ratios (β-ratios) for a more effective identification of volcanic ash in moist atmospheres [26]. Other authors [27] have implemented a set of fixed threshold tests on SEVIRI data to identify volcanic ash emitted by the Eyjafjallajökull volcano. An efficient three-band technique was developed to monitor ash clouds in several geographic areas, integrating two different spectral tests on brightness temperature differences *BT10.8 − BT12.0* and *BT8.7 − BT10.8* (which is based on a signal measured at 8.7 µm and 10.8 µm wavelengths) to reduce artifacts compared to the BTD technique [28]. Principal component analysis (PCA) was used with good results to identify ash-affected areas on MODIS data [29], and the potential of the Saharan Dust Index [30] in detecting volcanic ash was also assessed, evaluating its possible usage as a tool that is complementary to other established satellite-based procedures [31].

Those and other recent methods [32,33] have shown that improvements in ash detection from space are definitively possible. Nonetheless, the effective identification of volcanic ash over a wide range of observational and plume conditions (e.g., in the presence of underlying, overlying or shrouding clouds; water-rich plumes and/or with a low amount of ash) still represents a challenge. In addition, the use of empirical thresholds whose efficiency depends on several factors (e.g., viewing angles, particle size, and level of SO_2_ absorption) and/or the requirement of ancillary data represent important factors to take into account when the operational monitoring of ash clouds is required.

Hence, single ash-detection techniques show relative pros and cons depending on the way they are designed, on the spectral bands used, and in terms of the observational conditions they are applied to. Therefore, the combination of independent methods should, in principle, improve ash-detection capabilities although, to our knowledge, this has never actually been assessed and exploited so far.

With this aim, in this work we analyze and compare two advanced and completely independent satellite-based ash-detection techniques (described in detail in Section 3.1 and Section 3.2, respectively) in order to assess their possible joint usage in supporting operational ash monitoring.

## 3. Methods

### 3.1. RST_ASH_ Algorithm

RST_ASH_ is an unsupervised change-detection scheme, based on the general robust satellite techniques (RST) approach [34], which analyses relative rather than absolute signal variations in order to identify ash clouds by satellite. 

Specifically, multi-year time series of homogeneous cloud-free satellite records (i.e., acquired in the same month at same overpass times) are processed to generate the spectral reference fields of temporal mean and standard deviation, describing the “normal” behavior of the signal. To identify ash pixels, two local variation indices are then computed and used jointly. They are based on the Absolutely Local Index of Change of the Environment (ALICE) proposed by [34]:

(1) $${⊗}_{V}\left(x,y,t\right)=\frac{\left[V\left(x,y,t\right)-\mu_{V}\left(x,y\right)\right]}{\sigma_{V}\left(x,y\right)}$$

In Equation (1), *V*(*x*,*y*,*t*) is the satellite signal measured at time *t* (i.e., overpass time) and place (*x*,*y*) while *µ_V_*(*x*,*y*) and *σ_V_*(*x*,*y*) represent the temporal mean and standard deviation of the same signal measured in unperturbed conditions, respectively.

The RST_ASH_ algorithm uses the ⊗_Δ*TIR*_(*x*,*y*,*t*) and ⊗*_MIR−TIR_*(*x*,*y*,*t*) indices to detect volcanic ash. The first exploits the aforementioned reverse absorption behavior shown by volcanic ash in the split window bands (i.e., *V* = ∆*TIR* = *BT10.8* − *BT12.0*). The second takes into account the reflectance of ash clouds in the MIR band, as well as their different behavior in the MIR and TIR bands at night-time, investigating the brightness temperature difference between the signal measured in the SEVIRI channels centered at 3.9 µm and 10.8 µm wavelengths (i.e., *V* = *MIR* − *TIR* = *BT3.9* − *BT10.8*).

In more detail, negative values of ⊗_Δ*TIR*_(*x*,*y*,*t*) are expected in the presence of ash clouds, along with positives values of ⊗*_MIR_*_−*TIR*_(*x*,*y*,*t*) varying on the basis of illumination conditions (i.e., night-time/daylight). Since RST_ASH_ is tunable, we can use different cutting levels of the ⊗_Δ*TIR*_(*x*,*y*,*t*) index to discriminate regions with a different probability of ash presence in the atmosphere.

RST_ASH_ was tested for comparison with other well-established ash/dust detection methods [8,10] showing performance comparable to water vapor-corrected BTD procedures [35] despite some limitations widely discussed in previous works [36,37,38].

To improve the capability to discriminate ash from weather clouds, an optimized RST_ASH_ configuration has recently been developed [12]. As shown in previous studies, water and ice clouds generally exhibit a reflectance in the visible band (i.e., at around 0.65 µm) that is higher than pure volcanic ash of the same optical depth, with the only exception being for very fine ash [26]. The optimized daytime RST_ASH_ takes into account this different spectral behavior in the visible band computing the ⊗*_VIS_*(*x*,*y*,*t*) index, i.e., analyzing the signal measured in the SEVIRI channel 1 (0.56–0.71 µm). This index is less effective in detecting ash clouds, since their reflectance depends on several factors (e.g., particle size, ash content) as well as on illumination and viewing conditions [23]. However, once integrated within the RST_ASH_ scheme (i.e., used in combination with the two local variation indices described above) it may increase the filtering capabilities of meteorological clouds on daytime scenes [12]. Similarly, an additional local variation index based on a 10.8 µm TIR signal could be used for the same purpose in night-time conditions. However, since the RST_ASH_ algorithm integrating this index was not fully tested, we use here the following configuration to detect ash clouds by means of SEVIRI data:

*Daytime:* ⊗_Δ*TIR*_(*x*,*y*,*t*) < −2 AND ⊗*_MIR−TIR_*(*x*,*y*,*t*) > 1 AND ⊗*_VIS_*(*x*,*y*,*t*) < 3 (2)

*Night-time*: ⊗_Δ*TIR*_(*x*,*y*,*t*) < −2 AND ⊗_*MIR−TIR*_(*x*,*y*,*t*) > 1 (3)

In addition, we have integrated a spatial noise-reduction filter within the RST_ASH_ process to remove occasional patches of speckle (i.e., “salt and pepper” noise), similarly to the London VAAC method (see next section).

### 3.2. London Volcanic Ash Advisory Centers (VAAC) Ash-Detection Method

The detection scheme employed at the London VAAC uses five detection steps explained in full detail in [6]. The first test uses a straightforward brightness temperature difference (BTD) test, whereby a pixel is given a definite positive ash-detection flag if the observed BTD between the 10.8 μm and 12.0 μm channels is more negative than −2 K:

*BT10.8* − *BT12.0* < −2.0 K, definite detection (4)

Test 2 is a three-channel BTD which was developed during the Eyjafjallajökull eruption in 2010. In this test, a pixel is given a tentative ash-detection flag (see below) if:

(*BT10.8* − *BT12.0*) + (*BT10.8* − *BT8.7*) < 1.5 K, tentative detection (5)

Test 3 provides a tentative detection flag if the 2-channel BTD in Equation (4) is less than or equal to −0.7 and greater than or equal to −2.0.

Test 4 is used to remove any false alarms introduced by Tests 2 and 3, and is based on the work described by [39] using the concept of “β-ratios” as described by [40]. In this approach, effective ash-cloud emissivities are calculated from the observed radiances at 8.7 μm, 10.8 μm and 12.0 μm, and these are used to construct ratios of effective absorption optical thickness between pairs of channels. For example, the β-ratio for the pair of channels at 8.7 μm and 10.8 μm is given by:

(6) $$\beta (8.7,10.8)=\frac{\ln(1-\varepsilon (8.7))}{\ln(1-\varepsilon (10.8))}$$

where *ε* is the cloud emissivity in the 8.7 μm and 10.8 μm channels which is derived from the clear-sky and overcast radiances calculated from the RTTOVS (Radiative Transfer for TOVS) radiative transfer model and at an assumed radiative emission height [6].

The beta ratio for 12.0 relative to 10.8 is also calculated and the following thresholds are used to remove pixels which are thought not to be ash:

(7) $$\begin{array}{l} \beta \left(8.7,10.8\right)\leq 0.7\\ or\\ \beta \left(8.7,10.8\right)\geq 1.2\\ or\\ \;\;\;\;\beta \left(12.0,10.8\right)\;>4.2645-5.823\beta \left(8.7,10.8\right)+2.446\beta {\left(8.7,10.8\right)}^{2} \end{array}$$

Test 5 is a 3 × 3 spatial coherence test where six or more of the pixels have to contain ash for the pixel to be positively identified as ash-contaminated. This test is used to remove the “salt and pepper” noise that can be found with these thresholding techniques.

## 4. Data

### 4.1. Meteosat Second Generation (MSG) Spinning Enhanced Visible and Infrared Imager (SEVIRI)

**Figure 1:**
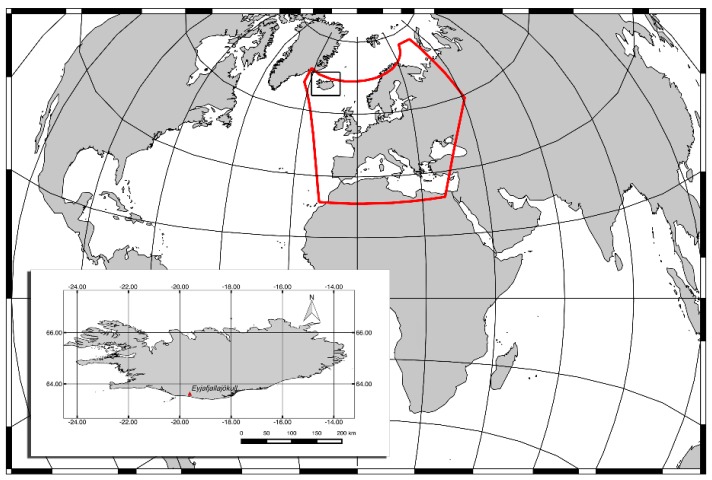
Geographic Earth map in azimuthal equidistant projection. The region investigated in this work is highlighted by the red polygon; the location of Eyjafjallajokull volcano is indicated within the panel at the bottom-left of the image.

SEVIRI is a scanning radiometer providing data in 12 different spectral bands spanning the visible and thermal infrared. The sensor offers a spatial resolution of about 3 km at the sub-satellite point in the standard channels and of 1 km in the high-resolution visible (HRV) channel. The HRV channel covers half of the full disk in the east-west direction and a full disk in the north–south direction [41]. These features along with the temporal resolution of 15 min make this sensor particularly suited to detect and track ash clouds from space in an operational framework. In this work, a subset of SEVIRI scenes covering the geographic area reported in red in Figure 1 and acquired in April and May 2010 are analyzed to evaluate the algorithm performance in different illumination conditions.

RST_ASH_ implementation on SEVIRI data is generally performed integrating the standard EUMETSAT (European Organization for the Exploitation of Meteorological Satellites) Cloud Mask (CLM) product [42,43] and the OCA (one-channel cloudy radiance-detection approach) method [44] (a RST-based cloud-detection scheme). However, since the CLM product is not generated for the whole Earth-disc coverage of SEVIRI, being the MPEF (meteorological products extraction facility) processing area defined as 65 degrees geocentric angle around the sub-satellite point [45], and Iceland is located at the margin of the MPEF processing area, in this study only the OCA method was implemented within the elaboration chain. This implies that residual clouds may contaminate the spectral reference fields, possibly affecting the RST_ASH_ results.

Regarding the London VAAC method, which was specifically designed and developed for SEVIRI, it does not require a multi-temporal analysis. Thus, the same procedure fully described in [6] was used and applied over the SEVIRI multi-spectral images shown in Section 5. 

### 4.2. EOS/Aqua Atmospheric Infrared Sounder (AIRS) 

Among the satellite instruments, the atmospheric infrared sounder (AIRS) may provide an important contribution for investigating volcanic ash in the infrared spectral region. AIRS is a hyperspectral IR sensor that has been orbiting on EOS/Aqua spacecraft since May 2002, providing high spectral-resolution records from 649–1136, 1217–1613 and 2169–2674 cm^−1^, with a spatial resolution of 13.5 km at nadir [46]. 

Some algorithms and products have been specifically developed to detect airborne ash/dust by means of AIRS data [47,48,49]. In this work, we compare SEVIRI ash detections to those independently performed by the AIRS dust-detection algorithm (DDA), whose information are stored as a layer (dust score) within the Level 1B AIRS data products. The latter are distributed online (in a standard digital format) through the NASA (National Aeronautics and Space Administration) EarthData GES DISC (Goddard Earth Science Data Information Services Center) portal [50].

The DDA night-time/daytime products (whose advantages and limitations are widely discussed in [49,51]) are independently generated using five AIRS channels in the 10 µm atmospheric window region, computing a set of brightness temperature differences and applying a number of tests for generating a summed score. AIRS pixels are flagged as ash/dust-affected if the score exceeds a certain threshold, which can assume different values for ocean and land regions. Suggested thresholds for the summed score index range from 360 (in case of desert regions) to 380 (for oceans) [49]. Here, we consider only the threshold value of 380, since most of the volcanic ash emitted during the Eyjafjallajökull eruption dispersed over the sea regions where the DDA product generally performs very well [51].

## 5. Results

### 5.1. Qualitative Comparison of SEVIRI Ash Products

In this section, we show a number of ash maps generated using the RST_ASH_ and London VAAC methods. Those maps are analyzed both separately and in an integrated manner in order to assess possible improvements resulting from their joint use. 

Both night-time (i.e., 00:00 UTC) and daytime (i.e., 06:00 and 12:00 UTC) ash maps are analyzed, in order to investigate how much the different illumination conditions may affect the outputs of algorithms used.

#### 5.1.1. April 2010

During the first explosive phase of the Eyjafjallajökull eruption (i.e., 14–18 April 2010), when very fine ash was emitted [52], the water-rich volcanic plume [53] moved towards Europe reaching an altitude of 5–10 km because of strong upper-level winds [54]. During the second phase of the eruption (18 April–4 May 2010), the explosive activity was relatively weak, the ash particles were coarser, and some water vapor affected the plume [53] extending up to 3–5 km above sea level (a.s.l.) [52].

**Figure 2:**
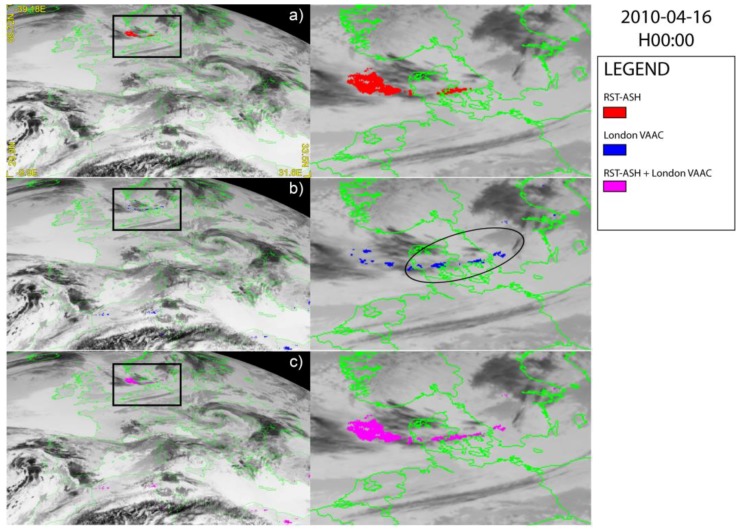
Spinning enhanced visible and infrared imager (SEVIRI) ash maps of 16 April 2010 at 00:00 UTC over-plotted on the SEVIRI channel IR 10.8 µm; (**a**) RST_ASH_; (**b**) London VAAC; (**c**) combined algorithms. On the right side of each panel the zoom of detected plume is shown.

**Figure 3:**
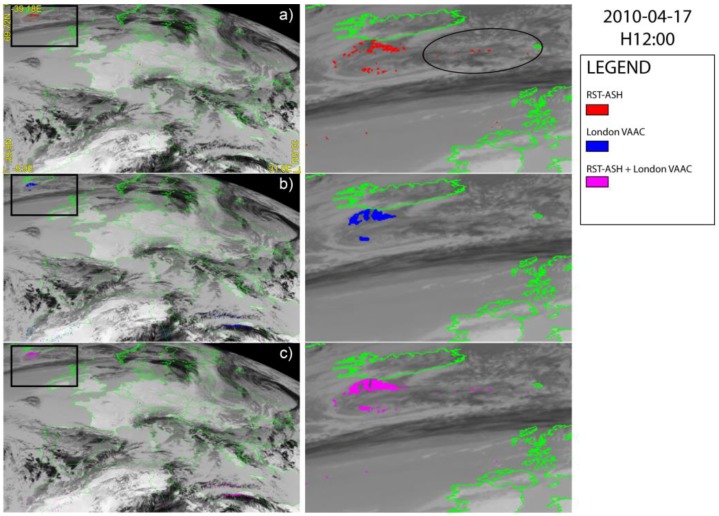
SEVIRI ash maps of 17 April 2010 at 12:00 UTC over-plotted on the SEVIRI channel IR 10.8 µm; (**a**) RST_ASH_; (**b**) London Volcanic Ash Advisory Center (VAAC); (**c**) combined algorithms. On the right side of each panel the zoom of detected plume is shown.

**Figure 4:**
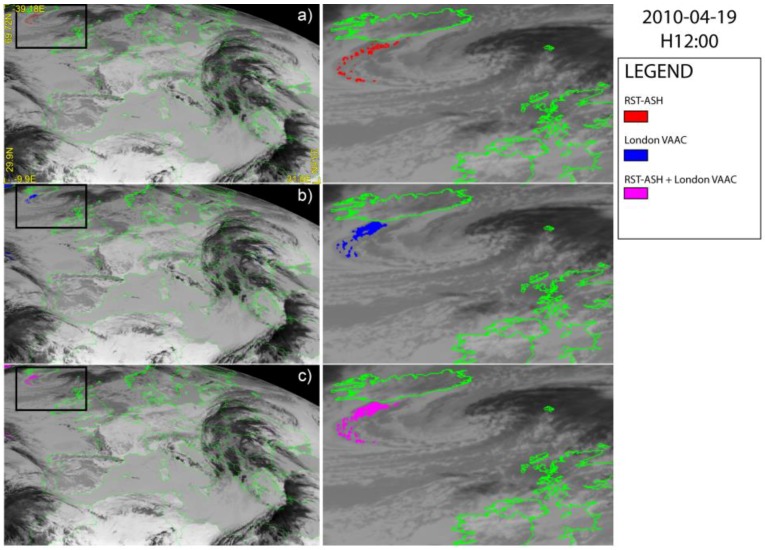
SEVIRI ash maps of 19 April 2010 at 12:00 UTC over-plotted on the SEVIRI channel IR 10.8 µm; (**a**) RST_ASH_; (**b**) London VAAC; (**c**) combined algorithms. On the right side of each panel the zoom of detected plume is shown.

Figure 2, Figure 3 and Figure 4 show a set of ash detection maps generated using SEVIRI data acquired in April 2010, over-plotted on the TIR SEVIRI image (channel IR 10.8 µm). Looking at Figure 2a,b, Figure 3a,b and Figure 4a,b, showing the ash maps produced by the single algorithms (Figure 2a, Figure 3a and Figure 4a report the RST_ASH_ maps while Figure 2b, Figure 3b and Figure 4b the London VAAC ones), it can be noted that both methods were capable of identifying volcanic ash, generating only some false positives having, in general, a low impact in terms of detection reliability. Those pixels were in fact always located very far from the source (e.g., North African coasts), and can be easily discriminated from the ash affected ones. On the other hand, both methods show some limitations in accurately mapping the ash plume extent, owing to observational conditions (e.g., the presence of meteorological clouds) or plume features (e.g., optical depth).

In particular, Figure 2 displays the ash maps of 16 April at 00:00 UTC, with the RST_ASH_ product displayed on the top (Figure 2a) and the London VAAC ash map shown in the middle (Figure 2b). As can we see, both methods detected an ash plume affecting part of the North Sea and moving east toward Denmark (see red and blue pixels within the black square magnified area on the right side of the same figure). Nevertheless, according to RST_ASH_ the ash covered a larger area over the sea than the London VAAC method (see Figure 2a), although the latter indicated that the volcanic plume was more elongated in the north-east direction (see pixels within the ellipse in Figure 2b; right side of the figure). Combining the maps provided by those independent ash-detection methods produces the ash plume shown in Figure 2c, which seems to have a more plausible spatial continuity of the ash-affected areas.

Figure 3 displays the daytime ash maps of 17 April at 12:00 UTC, showing the presence of an ash plume that was probably only partially identified by satellite, because of the thick cloud coverage affecting northern Europe (see the TIR image used as a background). It is worth noting that less-evident differences characterized the two ash products compared to maps of Figure 2. In more detail, the London VAAC method (Figure 3b) detected a higher number of ash pixels than RST_ASH_ (Figure 3a) close to Iceland’s coast, better detailing the proximal portion of the plume.

On the other hand, RST_ASH_ considered the ash cloud as slightly more extended in the easterly direction (see ash pixels detected over the region indicated by the ellipse in Figure 3a). Thus, even in this circumstance, the ash plume was better described integrating outputs of the two SEVIRI products (see Figure 3c).

Concerning the second phase of the Eyjafjallajökull eruption, Figure 4 displays the SEVIRI ash maps of 19 April at 12:00 UTC. The figure shows that the London VAAC method defined the 2D distribution of the proximal part of the plume in a more efficient way than RST_ASH_ (Figure 4a), despite a number of artifacts affecting the scene (e.g., see blue pixels at upper-left corner of Figure 4b). Nonetheless, the combination of outputs from the methods used allowed us to map the plume in a more accurate way (see Figure 4c) than with single-ash products (e.g., see Figure 4b).

#### 5.1.2. May 2010

**Figure 5:**
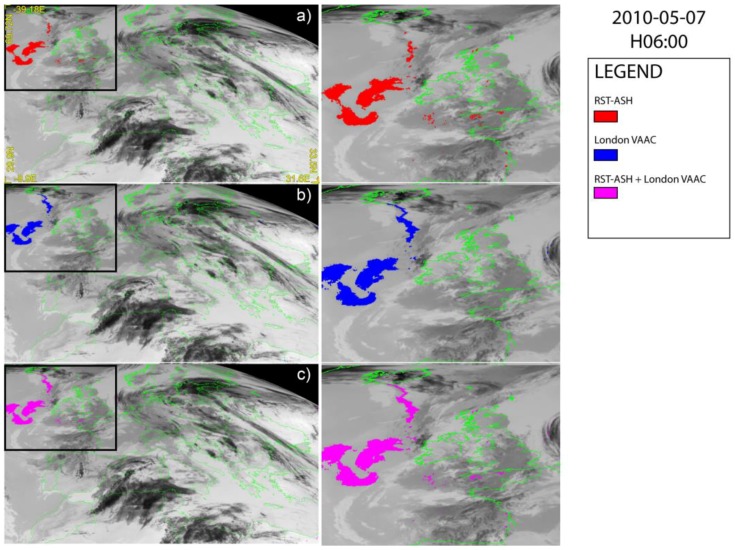
SEVIRI ash maps of 7 May 2010 at 06:00 UTC over-plotted on the SEVIRI channel IR 10.8 µm; (**a**) RST_ASH_; (**b**) London VAAC; (**c**) combined algorithms. On the right side of each panel the zoom of detected plume is shown.

During the third phase of the Eyjafjallajökull explosive eruption which began on 5 May 2010 [55] significant amounts of ash and pumice were injected into the atmosphere with the volcanic plume, which did not show evidence of water vapor [53], rising up to 10 km a.s.l. [52]. Figure 5 displays the RST_ASH_ and the London VAAC maps of 7 May at 06:00 UTC.

The figure shows that the London VAAC method (Figure 5b) performed better than RST_ASH_ (Figure 5a) in mapping the proximal region of the plume. Nevertheless, compared to the ash maps of Figure 3, the two algorithms performed in a more similar way in the distal region of the plume, despite some possible artifacts affecting the RST_ASH_ product (see red pixels located south of England in Figure 5a). Hence, integrating outcomes of ash detection (Figure 5c), the plume did not show any significant difference compared to that of Figure 5b. It seems, in fact, that the combination of two ash products did not greatly increase the performance of ash detection in this case. 

**Figure 6:**
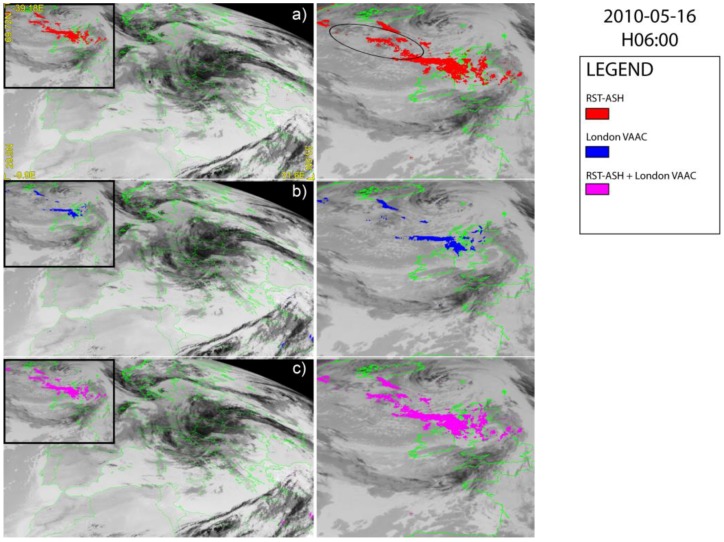
SEVIRI ash maps of 16 May 2010 at 06:00 UTC over-plotted on the SEVIRI channel IR 10.8 µm; (**a**) RST_ASH_; (**b**) London VAAC; (**c**) combined algorithms. On the right side of each panel the zoom of detected plume is shown.

Figure 6 displays the results for 16 May at 06:00 UTC showing that an ash cloud dispersing in the NW–SE direction affected the analyzed satellite scene. However, according to RST_ASH_ (Figure 6a) the ash plume was more extended than that indicated by the London VAAC (Figure 6b), which missed a number of ash pixels within the ellipse (Figure 6a), where the ash presence was confirmed by some independent satellite-based products [56]. Therefore, also in this circumstance, the combination of two algorithms (Figure 6c) did not significantly improve the results of ash detection from space (see Figure 6a for comparison).

### 5.2. Inter-Comparison of SEVIRI Ash Products

The results shown in the previous section (referring to last phase of the Eyjafjallajökull eruption, May 2010) indicate that, in contrast to what was observed in April 2010, ash maps generated combining outputs of London VAAC and RST_ASH_ algorithms did not provide evidence of a marked complementary behavior. 

Their joint usage did not introduce, however, significant noise and inaccuracies. Thus, it seems that even when the analyzed ash-detection methods do not show clear complementarities, their combination may still represent an advantage. In fact, the resulting ash maps could benefit from the performance of the algorithm working better (which is not necessarily always the same, as this study confirmed), without losing reliability.

In order to quantify the level of complementarities of the RST_ASH_ and London VAAC ash products, we summarize in Table 1 the performance of both algorithms, emphasizing their similarities and differences. In order to assess the performance of the detection algorithms, an expert mask, which classifies the pixels as ash and not ash, was created similar to [30]. The visual inspection of single-channel imagery, the two-channel BTD image, and red-green-blue (RGB) composite images allow for the creation of the expert mask. The London VAAC makes extensive use of the dust RGB composite images to qualitatively monitor volcanic ash. In the dust RGB composite image, the 12.0 µm minus 10.8 µm BTD is assigned to the red component of the image (with the intensity increasing as the BTD increases from −4 to +1 K), the 10.8 µm minus 8.7 µm BTD is assigned to the green component (over the range 0 to 15 K, with a gamma enhancement factor of 3.0), and the 10.8 µm brightness temperature is assigned to the blue component (over the range 261 to 289 K).

The dust RGB image was originally developed by EUMETSAT, who provide information and training material [42]. In more detail, by manually inspecting the imagery, detected pixels were assigned to belong to the “ash-confirmed” class (i.e., true positive) according to their: (i) distance from the source; (ii) shape and position; (iii) homogeneity compared to RGB-DUST products. Therefore, pixels not confirmed as ash were generally located far from the source (see previous section) resulting unambiguously ascribable to different causes as, for instance, residual cloud fronts and/or airborne dust coming from North Africa.

Table 1 shows that during the initial phase of the eruption (when the water vapor played an important role in the identification of the plume [57]) the number of ash pixels detected by two different algorithms was lower than during the last eruptive phase. In terms of accuracy of detection, the best scores achieved by the two methods were up to 99% for RST_ASH_ (on 16 April at 00:00 UTC) and about 71% for London VAAC (on 19 April at 12:00 UTC).

Regarding their complementarities, the “Total detections” column of Table 1 shows that the retrieved values were always greater than those obtained using each single algorithm alone. Thus, we can assess the added-value of the combination, by computing the number of detections with respect to the algorithm performing best. Using values reported in Table 1, such an indicator spans from ~3% (16 May) up to ~52% (17 April), quantifying the improvement achievable by a joint usage of the two different satellite-based products.

Along with the aforementioned differences, Table 1 shows that a number of common ash detections characterized the satellite scenes analyzed. They referred to “confirmed ash”, suggesting that when both algorithms identify the same areas those are very likely to be truly affected by volcanic ash. The latter is another advantage of the combined use of independent algorithms.

Concerning the last phase of the eruption, the best accuracy level of RST_ASH_ and London VAAC detections was achieved on 7 May at 06:00 UTC, and estimated around 95% and 98.5%, respectively (see Table 1). It is worth noting that the complementarities of RST_ASH_ and London VAAC methods was less evident during this eruptive phase (i.e., especially on 7 May when the two algorithms behave in a very similar way; see results shown in previous section). Nonetheless, the combination of ash products increased once again the efficiency of detection, as confirmed by the added-value indicator, which was estimated around 19%. Therefore, even when the monitored volcano emitted a larger amount of ash and the plume was not water vapor-rich, ash maps benefited from the algorithm performing better between the two.

Table 2 provides a further confirmation of this evidence summarizing the exclusive and the common contributions to the ash-cloud mapping of both algorithms, with reference only to the confirmed detections. In particular, the table shows that although in some cases a single method identified the largest part of the ash plume (e.g., 16 April and 16 May), there were situations (i.e., 17 April) where the integrated application of the two algorithms significantly improved the mapping of the volcanic ash distribution.

Hence, the use of a merged ash product, i.e., combining outputs of RST_ASH_ and London VAAC methods, could effectively increase performance in detecting ash clouds.

In the following section, we assess the quality of this product by comparison with the AIRS DDA maps (see Section 4.2), quantifying differences/similarities in the identification of ash-affected areas from space during the different phases of the investigated eruption.

### 5.3. Quantitative Comparison of Merged SEVIRI Product and AIRS Dust-Detection Algorithm (DDA) Maps

**Figure 7:**
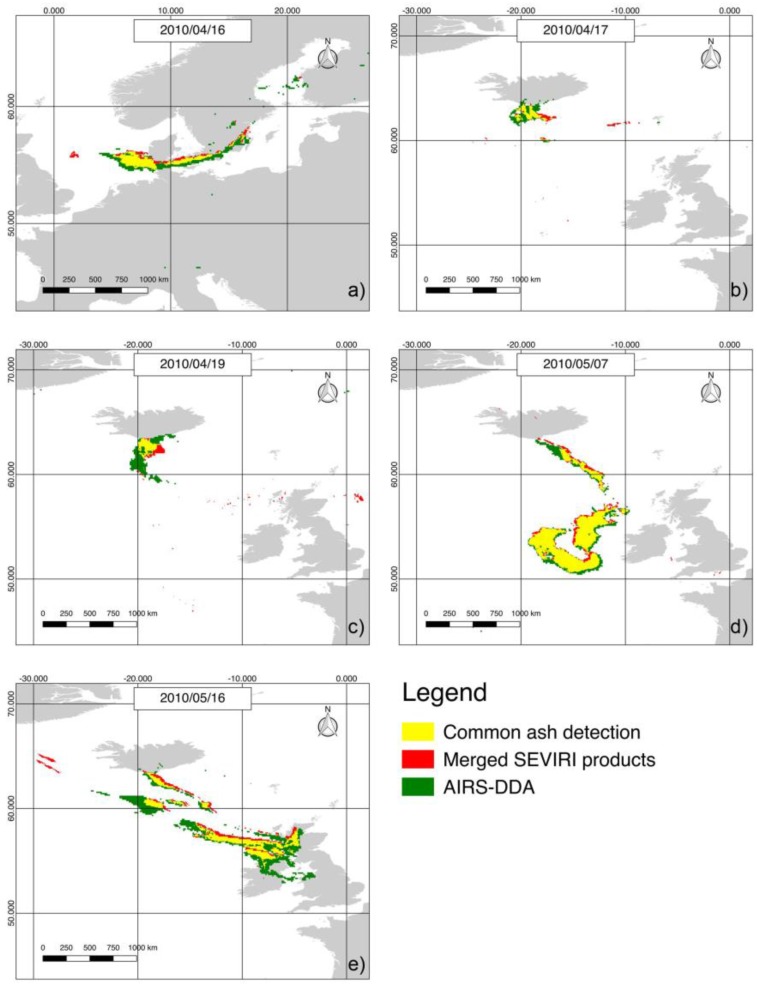
Comparison between merged SEVIRI ash product and AIRS dust-detection algorithm (DDA) maps (whose relative times are reported in Table 3) on the same geographic grid (LAT-LONG WGS 84).

To compare the merged SEVIRI-based ash product to the DDA maps, we selected the SEVIRI imagery that were closest in time to AIRS data (see Table 3). Within the free and open professional GIS application package QGIS [58], DDA and SEVIRI-based maps were re-projected and co-located on the same geographic grid (in this way most of geometric distortions were accounted for). Ash-affected areas were then quantitatively estimated by vectorizing raster data and comparing the obtained polygons, as shown in Figure 7.

Table 4 quantifies results of this investigation showing that the ash plume dispersed over a wider area during the last phase of the eruption (see maps of 7 and 16 May). Nevertheless, the area retrieved from AIRS DDA maps was always higher than that estimated from the merged SEVIRI product, revealing a different sensitivity to volcanic ash. In more detail, the table shows that the maximum value of the common ash-affected area (CA) was retrieved on 7 May when, despite some differences characterizing especially the proximal portion of the plume where it seems that the DDA performed better (see green area in Figure 7), results from SEVIRI and AIRS data were quite similar. On the other hand, the same panel reveals a slight spatial shift of the ash-affected area (see plume edges also on panels of 16 April and 16 May), which can be ascribed to residual geometric effects (e.g., parallax error) and/or to the minor temporal difference between AIRS and SEVIRI acquisitions (see Table 3). Regarding the minimum CA value in Table 4, it refers to maps of 17 and 19 April (see yellow areas on relative panels in Figure 7), when most of ash plume affected the sea region located close to Iceland.

Despite the aforementioned differences characterizing the analyzed satellite products, the ash-map integrating outputs of the RST_ASH_ and London VAAC methods provided information compatible with the DDA detections. It is worth noting that although the merged SEVIRI product probably underestimated some regions of the plume (see Table 4), it was capable of providing unique information over some sea area located far from the source (e.g., see 16 May in Figure 7), where the presence of volcanic ash appears plausible (see Section 5.1.2).

## 6. Discussion

In this study, we have compared two advanced satellite-based methods of ash detection, assessing their performance during the different phases of Eyjafjallajökull 2010 eruption.

The analysis of ash products generated from nighttime/daytime SEVIRI data has revealed some interesting differences in the identification of ash plumes. In particular, while the algorithm of London VAAC was in general more efficient close to the source of ash emissions, RST_ASH_ generally performed better in the distal region of the plume. The sensitivity of the London VAAC method relies upon the thresholds chosen for the final beta ratio test shown in Equation (7). The near source plumes are opaque and have a weak BTD signal but in beta ratio space the dense plumes are generally distinct from water and ice clouds. The distal regions have semi-transparent ash plumes, which have BTD signals that decline with ash concentration. In beta ratio space, the semi-transparent ash plumes with weak BTD signals look similar to low-level water cloud and the threshold chosen is a balance between sensitivity and false detection.

Regarding the RST_ASH_ algorithm, although it is self-adaptive and then capable of guaranteeing a good trade-off between reliability and sensitivity regardless of observational conditions [11,35], it is possibly affected by some limitations intrinsic in the use of BTD signal, whose values depend also on ash plume features. In particular, in the presence of very thick volcanic clouds (e.g., due to high concentrations of water droplets, ice crystals and large ash particles) the negative BTD signature of ash is generally weakened [25,26]. Hence, since the ⊗_Δ*TIR*_(*x*,*y*,*t*) index is based on that signal (see Section 3.1) the optically thicker plume regions (e.g., those closer to the source of ash emissions) may be only partially identified. On the other hand, RST_ASH_ is capable of performing better when ash clouds are more transparent, showing good performance in detecting the plume also over regions located far from the source.

Therefore, the results of this work have revealed a certain complementarities of RST_ASH_ and London VAAC algorithms, indicating that in some cases their combination could improve ash-cloud mapping capabilities by using SEVIRI data. 

To assess the quality of the merged RST_ASH_ product, we have performed a quantitative comparison with the well-established AIRS DDA maps. This analysis has shown that the merged SEVIRI product provided information about volcanic plumes, despite the underestimation of ash-affected areas, fitting with that provided by the AIRS maps. This result is particularly interesting considering that SEVIRI (although providing infrared data with a lower spectral resolution than AIRS) represents an important instrument for the operational monitoring of ash clouds from space, thanks to its features in terms of synoptic view and, above all, high temporal resolution. Further investigations are required, especially to assess the repeatability of the achieved results in different environmental conditions (e.g., geographic areas) as well as in the presence of various eruption styles and intensities (non-extreme events).

## 7. Conclusions

Although further investigations are required to assess differences and similarities of the two ash-detection techniques compared in this work, their combination could improve identification of ash-affected areas. Combination could be particularly helpful when observational/environmental and plume conditions (e.g., during the phreatomagmatic phase of the eruption) have a negative impact on the performance of single satellite-based ash products.

## Figures and Tables

**Table 1:** Number of ash pixels detected by RST_ASH_ and London VAAC methods on analyzed SEVIRI scenes. Both “total” (i.e., all the flagged) pixels and “confirmed” (i.e., those corroborated by the manual inspection; see text) are shown. First and hour columns report pixels flagged by the London VAAC and RST_ASH_ respectively. Column A reports pixels commonly identified by both methods, while columns B and C report the exclusive ash detections. The last column displays the total detections.

			London VAAC Detections	RST_ASH_ Detections	A. Common Detections	B. London VAAC Only Detections	C. RST_ASH_ Only Detections	Total Detections (A + B + C)
April 2010								
16 April 2010	00:00 UTC	Confirmed	302	912	140	162	772	1074
		Total	1258	922	140	1118	782	2040
17 April 2010	12:00 UTC	Confirmed	406	362	152	254	210	616
		Total	1025	446	152	873	294	1319
19 April 2010	12:00 UTC	Confirmed	370	230	105	265	125	495
		Total	670	237	105	565	132	802
May 2010								
7 May 2010	06:00 UTC	Confirmed	7567	6002	5563	2004	439	8006
		Total	7676	6451	5563	2113	888	8564
16 May 2010	06:00 UTC	Confirmed	2157	6252	1945	212	4307	6464
		Total	2591	6335	1945	646	4390	6981

**Table 2:** London VAAC and RST_ASH_ single (i.e., exclusive) and common contributions to the identification of ash plume during April and May 2010 eruptions.

Date [DD MM YYYY]	Time [hh:mm] (UTC)	RSTASH Contribution	VAAC Contribution	Common Contribution
April 2010				
16 April 2010	00:00	72%	15%	13%
17 April 2010	12:00	34%	41%	25%
19 April 2010	12:00	25%	54%	21%
May 2010				
7 May 2010	06:00	5%	25%	70%
16 May 2010	06:00	67%	3%	30%

**Table 3:** Times of compared SEVIRI and atmospheric infrared sounder (AIRS) data products.

DATE	AIRS (UTC; hh:mm:ss)	SEVIRI (UTC; hh:mm)
16 April 2010	01:17:24	01:15
17 April 2010	13:17:24	13:15
19 April 2010	13:05:24	13:00
7 May 2010	03:11:24	03:15
	03:17:24	
16 May 2010	03:05:25	03:15
	03:11:24	

**Table 4:** Estimates of ash-affected areas (including the common ones) from merged SEVIRI product and AIRS DDA maps. Values within parenthesis in the last column detail the percentage of common ash-affected areas in reference to the AIRS ones.

DATE	SEVIRI Areas (km^2^ × 10^3^)	AIRS Areas (km^2^ × 10^3^)	Common Areas (CA) (km^2^ × 10^3^)
16 April 2010	73.75	121.89	52.56 (43%)
17 April 2010	44.62	54.45	23.11 (42%)
19 April 2010	46.08	83.98	22.97 (27%)
7 May 2010	299.82	345.97	255.95 (74%)
16 May 2010	182.76	329.43	122.91 (37%)
